# Pregnancy-induced oxidative stress and inflammation are not associated with impaired maternal neuronal activity or memory function

**DOI:** 10.1152/ajpregu.00026.2024

**Published:** 2024-05-06

**Authors:** Jessica L. Bradshaw, E. Nicole Wilson, Jennifer J. Gardner, Steve Mabry, Selina M. Tucker, Nataliya Rybalchenko, Edward Vera, Styliani Goulopoulou, Rebecca L. Cunningham

**Affiliations:** ^1^Department of Pharmaceutical Sciences, University of North Texas Health Science Center, Fort Worth, Texas, United States; ^2^Department of Physiology and Anatomy, University of North Texas Health Science Center, Fort Worth, Texas, United States; ^3^Texas College of Osteopathic Medicine, University of North Texas Health Science Center, Fort Worth, Texas, United States; ^4^Lawrence D. Longo Center for Perinatal Biology, Departments of Basic Sciences, Gynecology and Obstetrics, Loma Linda University, Loma Linda, California, United States

**Keywords:** anxiety-like behavior, cognition, inflammation, oxidative stress, parity

## Abstract

Pregnancy is associated with neural and behavioral plasticity, systemic inflammation, and oxidative stress, yet the impact of inflammation and oxidative stress on maternal neural and behavioral plasticity during pregnancy is unclear. We hypothesized that healthy pregnancy transiently reduces learning and memory and these deficits are associated with pregnancy-induced elevations in inflammation and oxidative stress. Cognitive performance was tested with novel object recognition (recollective memory), Morris water maze (spatial memory), and open field (anxiety-like) behavior tasks in female Sprague-Dawley rats of varying reproductive states [nonpregnant (nulliparous), pregnant (near term), and 1–2 mo after pregnancy (primiparous); *n* = 7 or 8/group]. Plasma and CA1 proinflammatory cytokines were measured with a MILLIPLEX magnetic bead assay. Plasma oxidative stress was measured via advanced oxidation protein products (AOPP) assay. CA1 markers of oxidative stress, neuronal activity, and apoptosis were quantified via Western blot analysis. Our results demonstrate that CA1 oxidative stress-associated markers were elevated in pregnant compared with nulliparous rats (*P* ≤ 0.017) but there were equivalent levels in pregnant and primiparous rats. In contrast, reproductive state did not impact CA1 inflammatory cytokines, neuronal activity, or apoptosis. Likewise, there was no effect of reproductive state on recollective or spatial memory. Even so, spatial learning was impaired (*P* ≤ 0.007) whereas anxiety-like behavior (*P* ≤ 0.034) was reduced in primiparous rats. Overall, our data suggest that maternal hippocampal CA1 is protected from systemic inflammation but vulnerable to peripartum oxidative stress. Peripartum oxidative stress elevations, such as in pregnancy complications, may contribute to peripartum neural and behavioral plasticity.

**NEW & NOTEWORTHY** Healthy pregnancy is associated with elevated maternal systemic and brain oxidative stress. During postpregnancy, brain oxidative stress remains elevated whereas systemic oxidative stress is resolved. This sustained maternal brain oxidative stress is associated with learning impairments and decreased anxiety-like behavior during the postpregnancy period.

## INTRODUCTION

Motherhood requires widespread structural and functional adaptations in numerous organ systems during pregnancy and postpregnancy to ensure offspring development and suitable maternal caregiving behavior (1, 2). Specifically, the maternal central nervous system undergoes extensive remodeling during pregnancy and postpregnancy (2, 3). Clinical evidence suggests that the total brain volume of mothers decreases during pregnancy and recovers within 6 mo postpartum (3). Even so, maternal brain structural and functional dynamics have been shown to be region specific during pregnancy and postpregnancy (4–8), and these region-specific changes can be sustained for years after parturition (4, 5). Notably, a recent study revealed that pregnancy-associated reductions in gray matter volume within regions associated with cognition and mood are sustained through 2 yr postpregnancy in human cohorts (5).

Even though these pregnancy-associated physiological adaptations prepare mothers for the transition to parenthood, many of these adaptations are also associated with peripartum mental health disorders and crises, including peripartum depression, anxiety, and cognitive impairments (9–14). Indeed, ∼80% of pregnant women self-report lived experiences of cognitive impairment during pregnancy or postpregnancy (15), and pregnancy-associated cognitive declines including impairments in learning and working memory have been identified in women during pregnancy and postpregnancy (15–18). Moreover, women experiencing pregnancy complications such as preeclampsia, an obstetric disorder characterized by elevated systemic oxidative stress and inflammation, are at increased risk of developing cognitive impairments during pregnancy and later in life (19–21). Although there is growing evidence of structural brain fluctuations and cognitive impairments during pregnancy and postpregnancy, the underlying mechanisms contributing to maternal neuroplasticity and behavior remain elusive.

Reproductive status can impact numerous biological factors that modulate neuroplasticity and behavior (16). For instance, pregnancy-associated temporal patterns in hormone secretion modify the maternal brain and shape maternal behavior during pregnancy, parturition, and postpregnancy (22–24). Additionally, systemic fluctuations in the maternal immune response occur during the peripartum period and have been associated with alterations in neuroplasticity and maternal behavior (2, 25–28). Yet few studies have examined the localized neuroimmune response and its association with systemic inflammation during pregnancy and postpregnancy. Moreover, systemic inflammation is a prominent cause of oxidative stress, and oxidative stress impacts behavioral responses and neuronal processes including neurogenesis, migration, and synaptic pruning (29–32). Still, little is known regarding the impact of reproductive experience on inflammation and oxidative stress within brain regions associated with maternal cognitive functions.

The hippocampus and amygdala are two of the regions most vulnerable to oxidative stress and inflammatory insults, as these regions are reported as the first regions to undergo functional decline after exposure to stressors (32). In particular, the hippocampus is a highly plastic brain region that regulates learning and memory functions as well as the production of new neurons (33, 34). Within the hippocampus, CA1 neurons are critically involved in episodic recollective memory (35). Importantly, the hippocampus and amygdala are interconnected brain regions that cooperatively contribute to anxiety-like behavior (36). Of note, previous studies have demonstrated reductions in hippocampal volume and neurogenesis in women during pregnancy and postpregnancy (2, 16), as well as transcriptome alterations in the amygdala after parturition (37).

In this study, we used female rats with varying reproductive experiences (nulliparous, pregnant, and primiparous) to determine the impact of reproductive experience on maternal cognitive function and investigate oxidative stress and inflammation as modulators of maternal cognitive function. We hypothesized that healthy pregnancy transiently reduces learning and memory and these deficits are associated with pregnancy-induced elevations in inflammation and oxidative stress.

## MATERIALS AND METHODS

### Animals

All protocols were approved by the Institutional Animal Care and Use Committee (IACUC) of the University of North Texas Health Science Center (Protocol no. IACUC-2020-032). Protocols were performed in accordance with the National Institutes of Health *Guide for the Care and Use of Laboratory Animals*. All experiments were conducted with nulliparous (nonpregnant virgin, 9–11 wk old on arrival) or timed-pregnant (13–15 wk old on arrival) Sprague-Dawley female rats purchased from Envigo (Indianapolis, IN and Houston, TX). Pregnant rats arrived at the University of North Texas Health Science Center animal facilities on gestational day (GD) 10 (GD 1 = first day of copulatory plug; term = 22–23 days). Male rats were excluded from these studies because the focus of the research is the effects of pregnancy history (a female-specific condition) on maternal physiology.

Female rats were single-housed upon arrival under 12:12-h reverse light-dark cycles (lights off 0700, lights on 1900) in a temperature- and humidity-controlled environment. Animals were provided standard laboratory chow and water ad libitum. After 1 wk of acclimatization to the animal facilities, female rats were familiarized with operator handling to reduce stress responses during behavioral testing. This was a cross-sectional study, and four groups of rats were used: timed-pregnant rats at late gestation (PREG, GD 20–21) and a corresponding age-matched nulliparous group (NULLI-A, virgins) and timed-pregnant rats that were tested after they gave birth (primiparous, PRIMI, 1–2 mo after first pregnancy) and their corresponding age-matched nulliparous group (NULLI-B, virgins). A timeline and experimental design are illustrated in Supplemental Fig. S1 (see https://doi.org/10.6084/m9.figshare.25438894). NULLI-A and NULLI-B groups were not different in any outcome measure (*P* > 0.05), and their responses were collapsed into one group (NULLI). Rats assigned to the PRIMI group were allowed to give birth (term = 22–23 days), and pups were culled to six pups per litter within 48 h of delivery. Pups were weaned at postnatal day 28. Postpregnancy behavior testing of the PRIMI group began 1 wk after weaning to assess behaviors without the confounding effects of pregnancy- and lactation-associated hormonal fluctuations. The total number of animals used in this study was 31 female rats assigned as NULLI (*n* = 16), PREG (*n* = 8), and PRIMI (*n* = 7). All experiments were performed when female rats were 4–6 mo old.

### Behavioral Testing

Behavioral studies were conducted under red lighting over 2 days during the rodent’s active period (0800 to 1400). PREG and PRIMI rats were tested in separate cohorts alongside age-matched NULLI rats. Behavioral tests were used to assess hippocampus-associated memory (novel object recognition and Morris water maze) and anxiety-like behavior (open field exploration). Rats were placed in carriers and acclimated to the behavior testing room for 30 min before testing began. All testing equipment was thoroughly cleaned with 70% ethanol between animals. Behaviors were recorded with ANY-maze video tracking software (version 5.14; Stoelting Co.) for subsequent analysis by an investigator blinded to assigned groups.

#### Novel object recognition.

Novel object recognition was performed to assess short-term memory as previously described (38). Briefly, two objects of the same color, shape, and size were placed in adjacent corners at the top of the arena [24 in. (*W*) × 24 in. (*L*) × 12 in. (*H*)]. Animals were individually placed facing away from the objects in the bottom of the arena and allowed to explore the arena and objects for 5 min. After habituation to the arena and objects, animals were returned to their respective carriers to rest for 1 h. To assess short-term memory, one of the objects from the previous phase was placed back into the arena at its previous location (familiar object) and the other object was replaced in the same location with a novel object of different shape, color, and texture (novel object). After resting for 1 h, the animals were allowed to explore the arena and the two objects (1 familiar and 1 novel) for 3 min. Latency to initial movement, latency to novel object and familiar object, total contacts with the novel object and familiar object, object preference, and distance traveled during the test were examined.

#### Morris water maze.

To assess learning and spatial memory, the Morris water maze was performed as previously described (38, 39). Briefly, rats were trained to swim to a visible platform ∼1 cm above the water surface of an opaque pool (23–25°C) on *day 1* of the behavioral task. On *days 2–4* of training (learning phase), rats were trained to locate a submerged target platform within 90 s (3 trials/day), and each rat was allowed 20 s to sit on the located platform to observe visual cues placed on the walls to aid in the formation of spatial memory. On *day 5* (probe trial), the submerged target was removed and each rat was provided 30 s to swim to the target location and search for the platform. Learning performance on *days 2–4* was assessed by quantifying the latency and pathlength to the target as well as a learning index (40). Spatial memory was assessed during the probe trial on *day 5* by quantifying latency and pathlength to the target zone.

#### Open field.

Anxiety-like behaviors were assessed in an open field arena. Animals were individually placed in the bottom of the open arena [24 in. (*W*) × 24 in. (*L*) × 12 in. (*H*)] facing away from the open arena and allowed to explore for 5 min. The number of entries into the center of the open field, duration in the center, distance traveled in the center, and total distance traveled were assessed.

### Euthanasia and Tissue Harvest

Animals were anesthetized with 2–3% isoflurane and euthanized via decapitation during the animals’ active phase (0900–1100). All animals were euthanized within 4 days after the final day of behavior testing. Trunk whole blood was collected and plasma was isolated with EDTA-coated collection tubes (BD, catalog no. 367856) and centrifuged at 2,000 *g* for 10 min at 4°C. Freshly isolated plasma was aliquoted and stored at −80°C until further analysis. After euthanasia, brains were quickly removed, flash-frozen in 2-methylbutane (Millipore Sigma, catalog no. MX0760), and stored at −80°C until further analysis.

### Sample Preparation

Frozen brains were thawed in chilled 1× phosphate-buffered saline (Fisher, catalog no. BP399) and sliced into 1-mm coronal sections with a brain matrix (ASI Instruments, catalog no. RBM-4000C). Brain nuclei within the CA1 of the dorsal hippocampus (−5.30 mm from bregma) and basolateral and basomedial amygdala (−3.30 mm from bregma) were microdissected according to Paxinos and Watson’s brain atlas (41) with blunt 20-gauge needles attached to 1-mL syringes. Microdissected brain punches were stored at −80°C until further analysis. Frozen CA1 and amygdala microdissections were thawed in RIPA lysis buffer (VWR, catalog no. N653) containing (per 0.5 mL) 2.5 µL of Halt protease and phosphatase inhibitor (Thermo Scientific, catalog no. 78442), 1 µL of 0.5 mM ethylenediaminetetraacetic acid (EDTA; Thermo Scientific, catalog no. J15694.AE), and 1 µL of 0.5 mM dithiothreitol (DTT; Millipore Sigma, catalog no. 43815) and homogenized as previously described (42). Total protein concentrations in CA1 and amygdala homogenates were quantified with Pierce BCA Protein Assay (Thermo Scientific, catalog no. 23225).

### Plasma and Brain Cytokine Analysis

Plasma and dorsal hippocampal CA1 cytokines were assessed with a MILLIPLEX Rat Cytokine/Chemokine Magnetic Bead Panel (Millipore Sigma, catalog no. RECYTMAG-65K) customized for detection of proinflammatory (TNF-α, IL-6, IL-1β, IL-17A) and anti-inflammatory (IL-10) cytokines. Plasma samples were diluted 1:2 and CA1 lysates were diluted 1:10 in assay buffer before the assay was performed according to the manufacturer’s instructions. All samples, standards, and quality controls were plated in duplicate, and cytokines were measured on a Luminex 200 instrument using xPONENT software version 4.3 (Luminex Corporation, Austin, TX). Quality control values for each cytokine were within the range provided by the manufacturer.

### Circulating Oxidative Stress

The concentration of oxidized proteins in plasma samples was quantified with the OxiSelect Advanced Oxidation Protein Products (AOPP) Assay Kit (Cell Biolabs, Inc., catalog no. STA-318). Plasma samples were diluted 1:2 in assay buffer before the assay was performed according to manufacturer’s instructions with modifications. Briefly, diluted plasma samples were analyzed with and without (water added in equal volume) the reaction initiator in the same plate to calculate plasma background readings at an optical density of 340 (OD_340_). Plasma background values were then subtracted from the respective plasma OD_340_ before AOPP concentrations were quantified with reference to the standard curve.

### Circulating Corticosterone

Plasma corticosterone levels were assessed with a MILLIPLEX Hormone Magnetic Bead Panel (Millipore Sigma, catalog no. MSHMAG-21K). Plasma steroid hormones were extracted by acetonitrile extraction methods according to manufacturer’s instructions. Briefly, 375 µL of acetonitrile (Fisher Bioreagents, catalog no. BP2405-1) was added to 250 µL of plasma sample to precipitate proteins. The precipitated solution was centrifuged at 17,000 *g* for 10 min, and 500 µL of supernatant was collected and dried by vacuum centrifugation. Dried pellets were suspended in 200 µL of assay buffer and stored at −20°C. MILLIPLEX immunoassay was performed according to manufacturer’s instructions with an 18-h overnight incubation. Samples, standards, and quality controls were plated in duplicate, and corticosterone concentrations were measured on a Luminex 200 instrument using xPONENT software version 4.3 (Luminex Corporation). Quality control values for corticosterone were within the range provided by the manufacturer.

### Western Blot Analysis

Homogenized amygdala (basolateral and basomedial) and CA1 samples were denatured in Laemmli buffer (Bio-Rad, catalog no. 161-0747) containing 10% β-mercaptoethanol (Fisher, catalog no. BP176) and heated to 95°C for 5 min. Equal volumes of denatured samples containing 20 µg of total protein were loaded onto 4–15% polyacrylamide gels [Bio-Rad, catalog no. 4561084 (CA1) or 4561086 (amygdala)] and resolved in Tris-glycine running buffer (Bio-Rad, catalog no. 1610771) at 25 mA for 1.5 h at room temperature. Resolved proteins were transferred to PVDF membranes at 80 V for 2 h at 4°C. Membranes were blocked for 1 h at room temperature with 5% nonfat milk in 1× Tris-based saline-Tween 20 (TBST; Thermo Scientific, catalog no. 28360). Membranes were incubated overnight at 4°C with primary antibodies probing Spectrin and enzyme-mediated degradation products (mouse anti-Spectrin, 1:1,000, Millipore Sigma, catalog no. MAB1622) and early growth response protein-1 (mouse anti-Egr-1, 1:100, Santa Cruz Biotechnology, catalog no. sc-515830) diluted in 1% nonfat milk-TBST solutions. For protein normalization, we used β-actin primary antibody diluted in 1% nonfat milk-TBST solution (1: 3,000, GeneTex, catalog no. GTX629630) and incubated for 1 h at room temperature. Membranes were washed in 10-min increments in TBST for 30 min before incubation with secondary antibody. Membranes were incubated for 1 h at room temperature with horseradish peroxidase-conjugated horse anti-mouse IgG solution (1:2,500, Cell Signaling, catalog no. 7074P2) diluted in 1% nonfat milk-TBST. Immunoreactive bands were visualized with a West Pico (Thermo Scientific, catalog no. 34580) or West Femto (Thermo Scientific, catalog no. 34095) enhanced chemiluminescence detection assay in a Syngene G:Box imager using GeneSys Image Acquisition software (version 1.5.2.0; Syngene). Band densitometry was quantified with ImageJ software (version 1.53t; National Institutes of Health). Total Spectrin (270 kDa) and enzymatic cleavage of Spectrin by calpain (150 kDa) and caspase-3 (120 kDa) were first normalized to β-actin (42 kDa) values before normalizing cleavage products to total Spectrin values. Egr-1 (54–58 kDa) values were normalized to β-actin values. Quantification of protein expression is presented as % NULLI, where samples are normalized to NULLI samples of the same cohort run in the same Western blot. Representative images of full Western blot membranes can be found in Supplemental Fig. S2 (see https://doi.org/10.6084/m9.figshare.23982444).

### Data and Statistical Analyses

Data and statistical analyses were conducted in Prism software (version 9.2; GraphPad). Outliers were identified and removed for each outcome measure by robust regression and outlier removal (ROUT) with a coefficient *Q* = 1%. Data distributions were tested for normality by Shapiro–Wilk test. Data determined to have non-Gaussian distributions were log transformed before application of parametric statistics. One-way ANOVA followed by Tukey’s multiple comparisons test was used to compare group differences unless otherwise noted. The relationship between inflammatory cytokines in the circulation and hippocampal CA1 was determined by Spearman correlation analysis. Spatial learning over the training period was assessed by two-way repeated-measures ANOVA with Sidak’s multiple comparisons test. Spatial learning indexes and memory were examined with unpaired *t* tests. The significance level was set to α = 0.05, and *P* ≤ 0.05 was considered significant. Values are expressed as means ± SD unless otherwise indicated.

## RESULTS

### Pregnancy-Associated Elevations in Systemic Inflammatory Cytokines Are Resolved during the Postpregnancy Period and Do Not Correlate with Hippocampal Inflammatory Cytokines during Pregnancy

**Figure 1.**
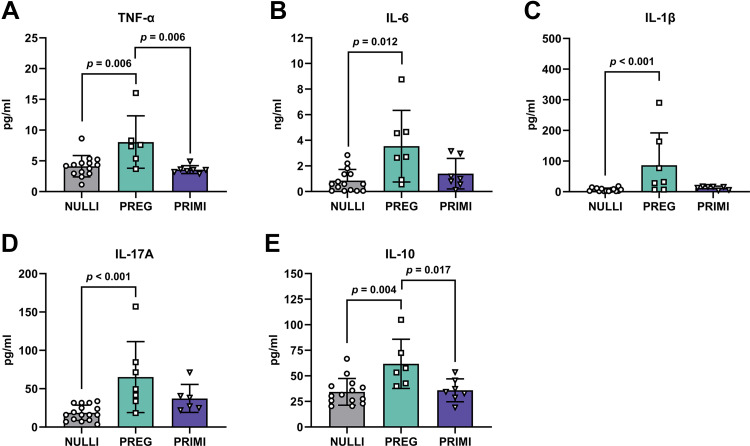
Circulating inflammatory cytokine profiles in healthy rats with various reproductive experiences: plasma concentrations of proinflammatory (TNF-α, IL-6, IL-1β, IL-17A) and anti-inflammatory (IL-10) cytokines. NULLI, nulliparous (*n* = 14–16); PREG, gestational day 21 (*n* = 6 or 7); PRIMI, primiparous (2 mo postpregnancy, *n* = 6 or 7). One-way ANOVA with Tukey’s multiple comparisons test, means ± SD. Outliers removed before analysis: TNF-α (*A*): NULLI = 2 (*n* = 14), PREG = 1 (*n* = 6); IL-6 (*B*): NULLI = 1 (*n* = 15); IL-1β (*C*): NULLI = 1 (*n* = 15); IL-17A (*D*): PRIMI = 1 (*n* = 6); IL-10 (*E*): NULLI = 2 (*n* = 14), PREG = 1 (*n* = 6).

**Figure 2.**
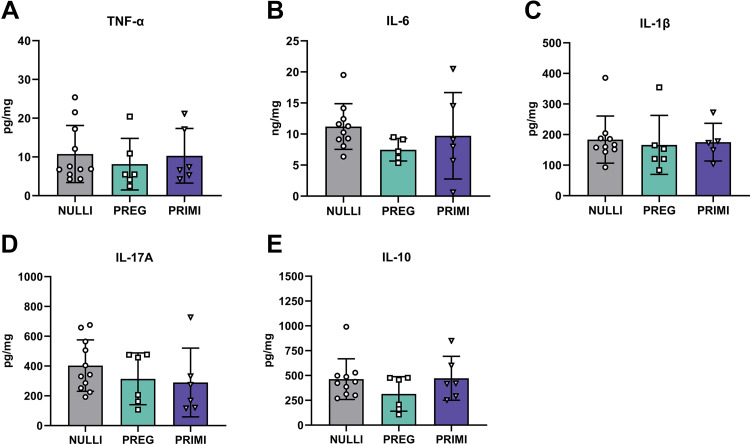
Inflammatory cytokine profiles in dorsal hippocampal CA1 of healthy rats with various reproductive experiences: concentrations of proinflammatory [TNF-α (*A*), IL-6 (*B*), IL-1β (*C*), IL-17A (*D*)] and anti-inflammatory [IL-10 (*E*)] cytokines within the CA1 of the dorsal hippocampus. NULLI, nulliparous (*n* = 10 or 11); PREG, gestational day 21 (*n* = 5 or 6); PRIMI, primiparous (2 mo postpregnancy, *n* = 5 or 6). One-way ANOVA with Tukey’s multiple comparisons test, means ± SD. Outliers removed before analysis: IL-6 (*B*): PREG = 1 (*n* = 5), NULLI = 2 (*n* = 10); IL-1β: NULLI = 2 (*n* = 10), PRIMI = 1 (*n* = 5); IL-17A: NULLI = 1 (*n* = 11); IL-10: NULLI = 2 (*n* = 10).

We observed elevated plasma proinflammatory (TNF-α, IL-6, IL-1β, IL-17A) and anti-inflammatory (IL-10) cytokines in pregnant rats during late gestation (GD21) compared with nulliparous rats (*P* ≤ 0.012; Fig. 1). Additionally, TNF-α and IL-10 plasma levels were reduced at 2 mo postpregnancy in primiparous rats compared with late gestation pregnant rats (*P* ≤ 0.017; Fig. 1). No significant differences were observed between nulliparous and primiparous rats in plasma levels of proinflammatory or anti-inflammatory cytokines (*P* > 0.05; Fig. 1). We next determined whether pregnancy-associated elevations in circulating cytokines were reflected in dorsal hippocampal CA1, which is a brain region that contributes to cognitive function (43). There were no group differences in proinflammatory or anti-inflammatory cytokines in the CA1 (*P* > 0.05; Fig. 2). Moreover, we did not observe any correlations in circulating plasma cytokines with CA1 cytokine levels (*P* > 0.05; Supplemental Table S1; see https://doi.org/10.6084/m9.figshare.23980191).

### Pregnancy Is Associated with Elevated Circulating and Hippocampal Oxidative Stress

**Figure 3.**
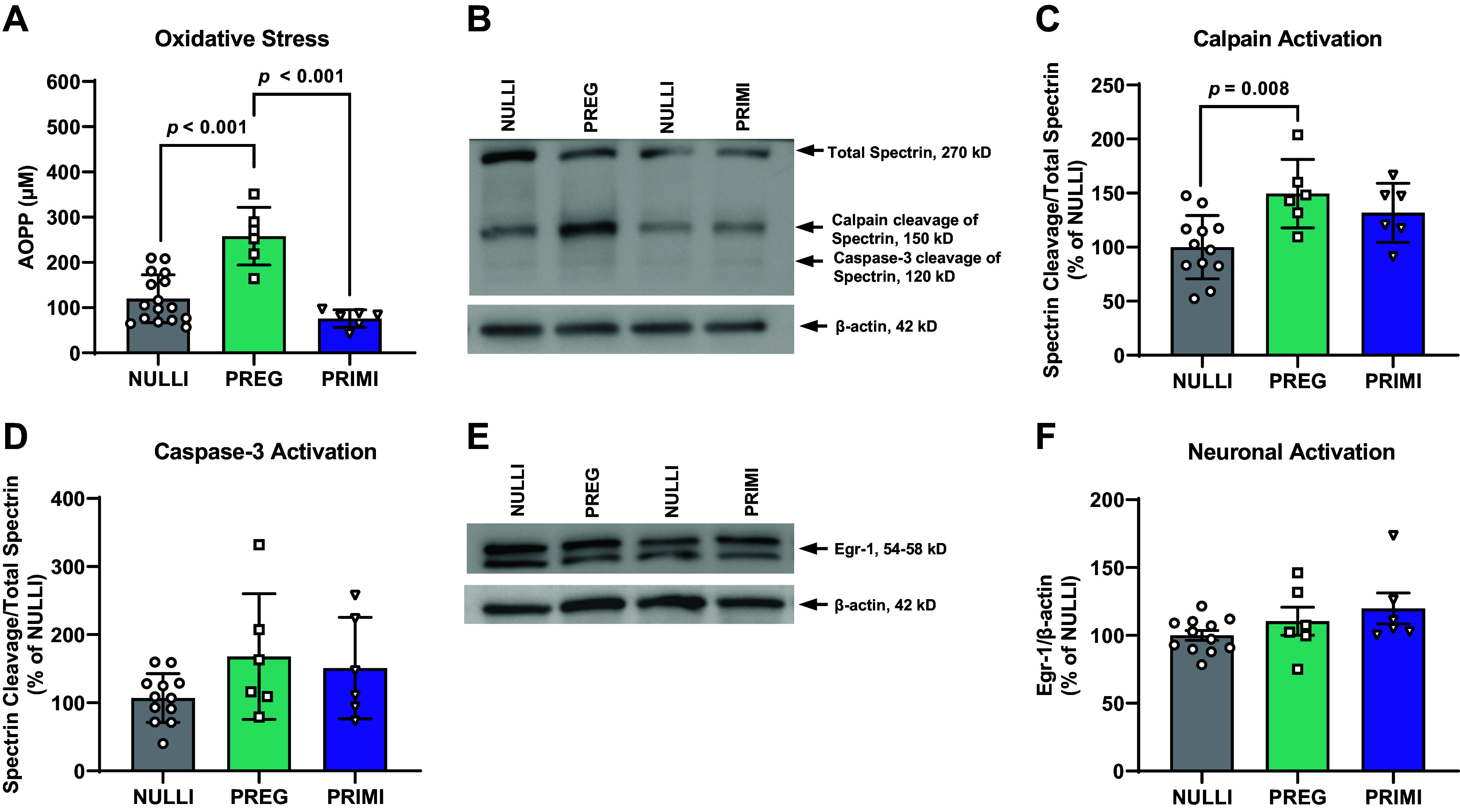
Circulating oxidative stress and cell activity in CA1 of dorsal hippocampus. *A*: plasma concentrations of advanced oxidation protein products (AOPP, measure of oxidative stress). *B*: Western blot image of enzyme-mediated cleavage of Spectrin (270 kDa), resulting in cleaved fragments at 150 kDa (calpain mediated) and 120 kDa (caspase-3 mediated) in CA1. *C*: quantification of calpain-mediated cleavage of Spectrin (measure of oxidative stress) in CA1. *D*: quantification of caspase 3-mediated cleavage of Spectrin (measure of apoptosis) in CA1. *E* and *F*: protein expression of early growth response protein-1 (Egr-1, neuronal activation marker) in CA1. NULLI, nulliparous (*n* = 12–16); PREG, gestational day 21 (*n* = 6); PRIMI, primiparous (2 mo postpregnancy, *n* = 6). Total Spectrin and cleavage products were first normalized to β-actin, and a proportion of cleaved product to total Spectrin was analyzed. Protein expression shown as % of NULLI. PREG rats were compared with age-matched NULLI rats run in parallel (*lane 1* in *B* and *E*), and PRIMI rats were compared with age-matched NULLI rats run in parallel (*lane 3* in *B* and *E*). NULLI control rats were collapsed into 1 group for group comparisons. One-way ANOVA with Tukey’s multiple comparisons test, means ± SD. No outliers removed before analysis.

Advanced oxidation protein products were elevated in the plasma of pregnant rats compared with nulliparous and primiparous rats (*P* < 0.001; Fig. 3*A*). Additionally, oxidative stress-associated calpain activation in the dorsal hippocampal CA1 was elevated in pregnant rats compared with nulliparous rats (*P* = 0.008; Fig. 3, *B* and *C*). We then determined whether there were any effects of pregnancy on markers of apoptosis and neuronal activation within the CA1. We did not observe any group differences in caspase-3 activation (*P* > 0.05; Fig. 3, *B* and *D*) or neuronal activation (*P* > 0.05; Fig. 3, *E* and *F*) within the dorsal hippocampal CA1.

### Primiparity Does Not Impair Recollective Memory during Pregnancy or Postpregnancy

**Figure 4.**
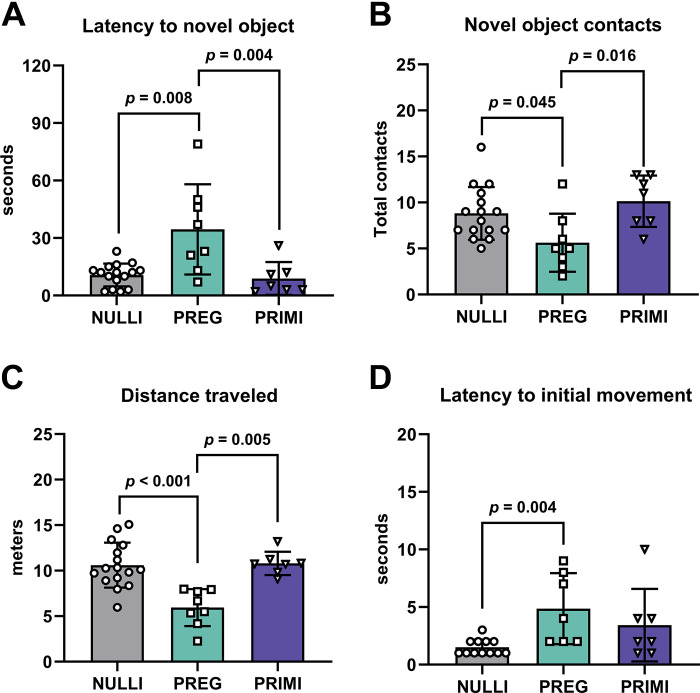
Recollective memory during novel object recognition behavior task. *A*: latency to initial contact with novel object. *B*: total contacts with novel object. *C*: total distance traveled during test. *D*: latency to initial movement during test. NULLI, nulliparous (*n* = 12–16); PREG, gestational day 20 (*n* = 7 or 8); PRIMI, primiparous (2 mo postpregnancy, *n* = 7). One-way ANOVA with Tukey’s multiple comparisons test, means ± SD. Outliers removed before analysis: latency to initial movement (*D*): NULLI = 4 (*n* = 12), PREG = 1 (*n* = 7).

Since we observed pregnancy-induced elevations in oxidative stress within the CA1 of pregnant rats, we determined whether CA1-mediated cognitive performance was affected, using the novel object recognition behavior task. Pregnant rats showed increased latency to initial contact with the novel object (*P* ≤ 0.004; Fig. 4*A*) and decreased total novel object contacts (*P* ≤ 0.045; Fig. 4*B*) compared with nulliparous and primiparous rats. However, pregnant rats also traveled less throughout the test compared with nulliparous and primiparous rats (*P* ≤ 0.005; Fig. 4*C*). Additionally, pregnant rats took longer to initiate movement in the behavior test compared with nulliparous rats (*P* = 0.004; Fig. 4*D*). Although there were no group differences in latency to initial contact of the familiar object (*P* > 0.05; Supplemental Fig. S3A; see https://doi.org/10.6084/m9.figshare.23982261), pregnant rats exhibited reduced total contacts with the familiar object compared with nulliparous and primiparous rats (*P* ≤ 0.025; Supplemental Fig. S3B). Furthermore, there were no group differences in object preference, as all groups spent more time investigating the novel object compared with investigating the familiar object (*P* > 0.05; Supplemental Fig. S3C).

### Primiparity Diminishes Learning during the Postpregnancy Period

**Figure 5.**
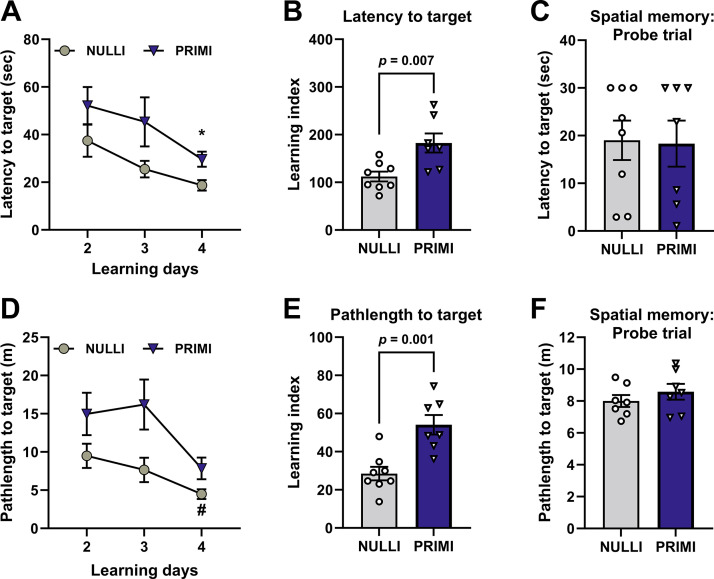
Learning indexes and spatial memory during Morris water maze. *A*: latency to target on learning *days 2–4* of behavior task. *B*: learning index calculated for each rat based on latency to target on learning *days 2–4.* A lower learning index represents better learning. *C*: spatial memory assessed via latency to target on *day 5* of behavior task. *D*: pathlength to target on learning *days 2–4* of behavior task. *E*: learning index calculated for each rat based on pathlength to target on learning *days 2–4*. A lower learning index represents better learning. *F*: spatial memory assessed via pathlength to target on *day 5* of behavior task. NULLI, nulliparous (*n* = 7 or 8); PRIMI, primiparous [1 wk after weaning (postpregnancy day 35), *n* = 7]. Two-way repeated-measures ANOVA with Sidak’s multiple comparisons test (*A* and *D*) or unpaired *t* test (*B*, *C*, *E*, and *F*), means ± SE. *P < 0.05 vs. PRIMI *day 4*; #*P* < 0.05 vs. NULLI *day 2*. No outliers removed before analysis.

To examine hippocampus-dependent spatial learning memory function, we performed the Morris water maze (Fig. 5) in age-matched nulliparous and primiparous rats 1 mo before performing the novel object recognition test (Fig. 4). Because of the additional stress of a swimming test, we did not test pregnant rats during late gestation in the Morris water maze to prevent additional stressors during pregnancy that could alter pregnancy outcomes. We observed main effects of parity (*P* = 0.009) and learning day (*P* = 0.012) with no interactive effects (*P* = 0.768) on latency to target during learning *days 2–4* (Fig. 5*A*). Additionally, primiparous rats exhibited increased latency to target compared with nulliparous rats on learning *day 4* (*P* = 0.048; Fig. 5*A*). When latency to target was assessed, nulliparous rats were better learners than primiparous rats during *days 2–4* of learning (*P* = 0.007; Fig. 5*B*). However, no group differences were observed when spatial memory was assessed on *day 5* of the Morris water maze (*P* = 0.914; Fig. 5*C*). Similar to latency to target, we observed main effects of parity (*P* = 0.009) and learning day (*P* = 0.014) with no interactive effects (*P* = 0.371) on pathlength to target on learning *days 2–4* (Fig. 5*D*). Nulliparous rats demonstrated shorter pathlengths to target on *day 4* compared with *day 2* of learning (*P* = 0.040), whereas primiparous rats did not exhibit shorter pathlengths as learning days progressed (*P* = 0.342; Fig. 5*D*). Similar to latency to target, nulliparous rats were better learners than primiparous rats when pathlength to target was assessed (*P* = 0.001; Fig. 5*E*), yet no group differences in spatial memory were observed on *day 5* when pathlength to target was evaluated (*P* = 0.372; Fig. 5*F*).

### Primiparity Reduces Anxiety-like Behavior during the Postpregnancy Period

**Figure 6.**
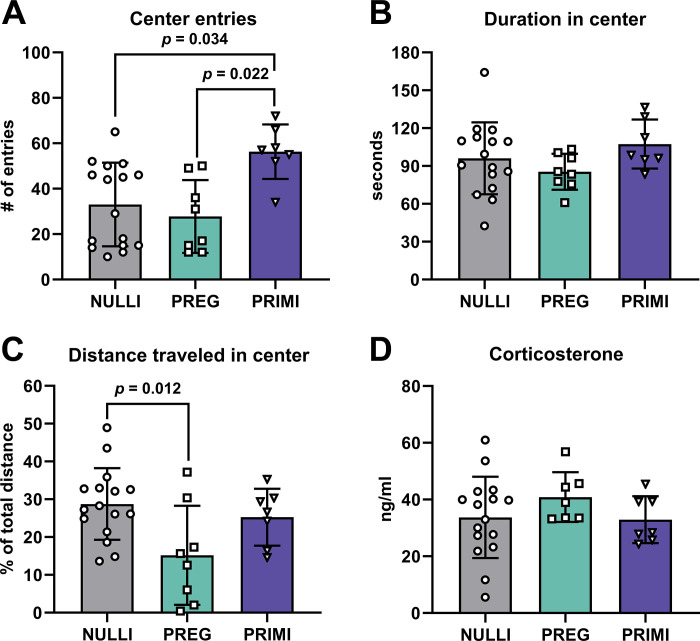
Anxiety-associated behavior during open field behavior task. *A*: total entries into the center of the open arena. *B*: duration in seconds in center of open area. *C*: distance traveled in the center of the open arena divided by the total distance traveled during behavior task. *D*: plasma concentrations of stress-associated steroid hormone corticosterone. NULLI, nulliparous (*n* = 16); PREG, gestational day 20 (*n* = 7 or 8); PRIMI, primiparous (2 mo postpregnancy, *n* = 7). One-way ANOVA with Tukey’s multiple comparisons, means ± SD. No outliers removed before analysis.

Primiparous rats exhibited an increased number of entries into the center of the arena compared with nulliparous and pregnant rats (*P* ≤ 0.034; Fig. 6*A*). However, primiparous rats were not spending more time in the center (*P* > 0.05; Fig. 6*B*) or traveling a greater distance in the center (*P* > 0.05; Fig. 6*C*) compared with nulliparous and pregnant rats, yet pregnant rats exhibited decreased distance traveled in the center compared with nulliparous rats (*P* = 0.012; Fig. 6*C*). Similar to the novel object recognition behavior task, pregnant rats traveled significantly less during the open field behavior test compared with nulliparous and primiparous rats (mean ± SD: NULLI: 16.53 ± 2.63 m, PREG: 12.62 ± 1.14 m, PRIMI: 19.60 ± 2.95 m; *P* ≤ 0.002). We observed no group differences in circulating levels of the stress hormone corticosterone (*P* > 0.05; Fig. 6*D*). Additionally, we observed no differences in the oxidative stress marker calpain-mediated Spectrin cleavage in the amygdala (*P* > 0.05; % NULLI, means ± SD: NULLI: 100.0 ± 32.19%, PREG: 108.9 ± 42.73%, PRIMI: 133.0 ± 69.01%; Supplemental Fig. S2: https://doi.org/10.6084/m9.figshare.23982444).

## DISCUSSION

In this study, we determined the impact of reproductive status on the association between markers of systemic and localized inflammation and oxidative stress with cognitive performance in rats. We found that systemic inflammation and oxidative stress are elevated during healthy rodent pregnancy but resolve in the postpregnancy period. In addition, maternal oxidative stress-associated enzymatic activity was elevated in the maternal dorsal hippocampal CA1 during pregnancy whereas inflammatory cytokines remained unchanged. Contrary to systemic oxidative stress, maternal CA1 oxidative stress levels were comparable in pregnant and primiparous rats at 2 mo postpregnancy. When assessing cognitive performance, we found that reproductive status did not impact recollective or spatial memory during pregnancy or postpregnancy. Although we did not observe memory impairments, we found that primiparous rats exhibit learning deficits and enhanced exploratory behavior in the center of an open arena in the postpregnancy period.

This is the first study to examine the effects of reproductive experience on the association between maternal systemic and localized brain inflammatory cytokines. We and others have observed increases in systemic inflammation during late gestation of rodent pregnancy (44, 45). Few studies have examined cytokine levels during postpregnancy and within the maternal brain during pregnancy and postpregnancy (46–49). Here, we found that inflammatory cytokine levels within the dorsal hippocampal CA1 were similar among rats of varying reproductive experience whereas plasma inflammatory cytokines drastically increased during pregnancy, suggesting that the maternal CA1 is protected from pregnancy-associated elevations in systemic inflammation. These findings are consistent with previous reports demonstrating no changes in maternal hippocampal IL-6 or IL-1β during late gestation or early postpregnancy in Sprague-Dawley rats (48). Similarly, a recent study by Duarte-Guterman et al. (47) observed no effect of reproductive experience (nulliparity, primiparity, biparity) on hippocampal inflammatory cytokine production at 30 or 240 days postpregnancy. Contrarily, a previous study by Haim et al. (46) revealed increases in IL-6 and IL-10 within the maternal dorsal hippocampus at 8 days postpregnancy in primiparous rats. Notably, these previous studies examined cytokine expression within the whole dorsal hippocampus, whereas the present study quantified cytokine expression specifically within the CA1 region of the dorsal hippocampus. Moreover, the present study and previous studies examined cytokine levels within the hippocampus at different reproductive states (i.e., late gestation or postpregnancy days 8, 30, 60, and 240). Taken together, reproductive experience may differentially impact specific regions of the maternal dorsal hippocampus (e.g., CA1, dentate gyrus, CA3) depending on reproductive status (early pregnancy vs. late pregnancy and early postpregnancy vs. late postpregnancy). Future studies examining cytokine expression levels in other hippocampus-associated brain areas, such as the amygdala and entorhinal cortex, during pregnancy and postpregnancy are warranted. Importantly, our findings of no correlations between systemic and localized cytokine expression levels highlights the restriction of pregnancy-associated systemic inflammation from infiltrating the maternal brain during pregnancy, as well as the limitation of plasma sampling as a proxy for maternal CA1 inflammation status.

This is also the first study to examine maternal systemic and hippocampal CA1 markers of oxidative stress during pregnancy and postpregnancy. We found that oxidative stress markers are elevated within the plasma (AOPP) and maternal CA1 (calpain enzymatic activity) during pregnancy. Importantly, systemic levels of oxidative stress declined in the postpregnancy period, whereas CA1 oxidative stress levels were comparable to levels observed during pregnancy, suggesting that pregnancy-associated elevations in oxidative stress may be longer-lasting in maternal tissues in comparison to the maternal circulation. Although oxidative stress-associated enzymatic activity was elevated within the maternal CA1 during pregnancy, we did not observe evidence of cell death (caspase-3 activation) or decreased neuronal activation. Collectively, the observed elevations in oxidative stress-associated markers occurred as a physiological response during healthy rodent pregnancy and suggest that the maternal CA1 is vulnerable to oxidative stressors during pregnancy; nonetheless, physiological elevations in maternal CA1 oxidative stress are not associated with impaired CA1 neuronal activity, apoptosis, or hippocampus-associated cognitive impairment. Similar to studies in maternal brain inflammation, future studies are needed to assess the impact of maternal oxidative stress in various brain regions associated with maternal behavior. Currently, there is growing evidence supporting the impact of pathophysiological elevations in oxidative stress during pregnancy, such as in gestational disorders, on fetal and offspring measures of brain oxidative stress and cognitive function (50), yet there is a paucity of studies examining the effect of elevated pregnancy-associated oxidative stress on maternal brain oxidative stress and cognitive function. Clinically, women who experience pregnancy complications associated with elevated oxidative stress, such as hypertensive disorders of pregnancy, are more likely to self-report cognitive impairments and have cognitive decline (19–21). Future studies interrogating oxidative stress within the maternal brain are needed to understand the role of maternal brain oxidative stress in physiological and pathophysiological reproductive states, including in animal models of gestational diabetes, hypertensive disorders of pregnancy, and maternal obesity.

When the impact of reproductive experience on recollective memory was assessed in the novel object recognition behavior task, pregnant rats exhibited increased latency to initial contact with the novel object and reduced overall novel object contacts. However, there were no differences among the groups in preference for the novel object. Of note, pregnant rats traveled less during the behavior task and were slower to initiate movement compared with both nulliparous and primiparous rats, which likely contributed to latency to the first interaction with the novel object and the total number of contacts over the test duration. Together, our data suggest that reproductive experience does not impact recollective memory. This finding is in agreement with previous observations of recollective memory in nulliparous, primiparous, and multiparous Long-Evans rats (51). Our findings also highlight the limitation of using the novel object recognition behavior task for assessing recollective memory in pregnant rats. Given that pregnant rats exhibit reduced locomotion, especially during late gestation, future studies could incorporate memory tasks that reduce mobility constraints, such as home cage-based recognition tasks that limit the arena size (52).

Similar to our findings on recollective memory, we also observed no effect of reproductive experience on spatial memory when assessing latency and pathlength to target during the probe trial of the Morris water maze. However, we did observe learning impairments in primiparous rats compared with nulliparous rats, as evidenced by increases in the learning indexes for latency to target and pathlength to target over the learning period (*days 2–4*). These findings in primiparous rats 35 days postpregnancy extend previous studies that demonstrated impaired spatial learning in the Morris water maze in primiparous rats 1–5 days postpregnancy (53). Of note, we incorporated the Morris water maze 1 mo before the novel object recognition behavior task in our experimental design to prevent confounds associated with battery testing (54, 55). Additionally, we conducted the Morris water maze 1 wk after weaning to also prevent any confounds related to weaning and lactation. Given prior reports indicating the potential of the Morris water maze to induce stress during rodent pregnancy (56), we did not examine spatial memory in pregnant rats. Nevertheless, previous studies have demonstrated that pregnant rats exhibit poorer spatial learning in the Morris water maze compared with nulliparous rats (56). It is postulated that impaired spatial learning is an adaptive response associated with nesting and preparing for parturition (56) and may extend into the postpregnancy period as mothers care for their young. Indeed, our findings reveal that spatial learning remains impaired at 35 days postpregnancy. Interestingly, a previous study by Pawluski et al. (57) revealed that spatial learning and memory are enhanced at 55 days postpregnancy compared with nulliparous rats, suggesting that previous pregnancy experience and time since pup separation have an impact on maternal spatial learning and memory.

Since pregnant rats were delayed in initial movement and demonstrated decreased total distance traveled during the novel object recognition behavior task, we examined anxiety-like behavior in an open field arena. Our findings demonstrate that primiparous rats exhibit reduced anxiety-like behavior in the open field behavior task, which is consistent with previous findings in young primiparous rats (58). However, primiparous rats did not spend more time or travel a greater distance in the center in the present study, revealing that center entries were abrupt. Contrarily, pregnant rats exhibited fewer center entries and shorter distances traveled in the center. Although this may be perceived as an anxiety-like behavior, we caution this interpretation given that pregnant rats also traveled less throughout the behavior task compared with nulliparous and primiparous rats, as we observed similarly in the novel object behavior task.

Because of group differences observed in anxiety-like behaviors, we investigated circulating stress hormones and oxidative stress within the amygdala, a brain region associated with anxiety-like behavior (59). We did not observe an impact of reproductive experience on plasma levels of the stress hormone corticosterone, which agrees with previous studies that examined corticosterone levels in primiparous rats during pregnancy and postpregnancy (60, 61). Of note, all rats were under brief isoflurane anesthetic before euthanasia, which may have contributed to reduced corticosterone levels in all rats (62). To our knowledge, this is the first study examining oxidative stress and cell death-associated enzymatic activity within the maternal amygdala. Our findings indicate that there were no group differences in oxidative stress-associated enzymatic activity or cell death within the amygdala. This is in contrast with our oxidative stress-associated observations in the CA1 during pregnancy, which further highlights the effects of reproductive experience on maternal brain oxidative stress as brain region specific.

Our study has numerous strengths including assaying systemic and localized cytokines and oxidative stress measures, conducting behavioral analyses during the rodent active cycle and outside of weaning in primiparous rats to prevent pup separation- and lactation-associated hormonal confounds, including complementary behavior tests to determine whether there were any pregnancy-associated confounds, and assessing hippocampus-associated maternal behaviors alongside region-specific molecular analyses. Even so, there are several limitations in this study that warrant further analyses. For instance, we did not assess the impact of reproductive experience on blood-brain barrier permeability, which may contribute to vulnerability of specific brain regions to oxidative stressors during physiological and pathophysiological pregnancies (63). Additionally, the reduced locomotive activity in pregnant rats during late gestation warrants the modification of memory and anxiety-like behavior tasks to accommodate reductions in overall movement. Alternatively, pregnant rats may require cognitive assessments earlier in gestation to eliminate reduced locomotion as a confounding factor. This is an important consideration for designing longitudinal studies, as specific time points in gestation would be limited for behavioral assessments. Moreover, we did not examine the impact of reproductive experience on specific cell types within the maternal CA1 that could be affected by systemic and local elevations in oxidative stress and inflammation, nor did we identify the specific source of oxidative stress within the hippocampus. Previous studies have revealed reductions in the density of brain-resident macrophages, microglia, during pregnancy and early postpartum (46, 49), but interactions of microglia with neurons and their role in modulating maternal behavior remain unclear. Importantly, neuronal plasticity and neuroexcitatory responses within the hippocampus are modulated by microglia and have been shown to both induce and be affected by oxidative stress (32, 64). Future studies could be aimed at understanding the impact of reproductive experience on cell-cell interactions, neuronal damage and plasticity, and oxidative stress production within various maternal brain regions during pregnancy and postpregnancy. Finally, our experimental design focused on late term and 2 mo postpregnancy, in which we observed elevations in oxidative stress within the maternal CA1. Future studies could incorporate temporal oxidative stress analyses spanning pregnancy and postpregnancy to provide a better understanding of fluctuations in oxidative stress within the maternal brain. Moreover, the impact of multiparity (2 or more previous pregnancies) on maternal oxidative stress, inflammation, and behavior in rats is largely unknown. In humans, multiparity is associated with increased risk of developing neurological disease with cognitive decline as well as increased rate of progression and severity of cognitive deficits (65–67). Thus, animal studies are needed to examine these associations and interrogate possible mechanisms and targets for intervention.

### Perspectives and Significance

Oxidative stress-associated markers, and not inflammatory cytokines, are elevated in the maternal CA1 during healthy pregnancy, revealing a vulnerability of the maternal hippocampal CA1 to oxidative stressors. Even so, elevations in physiological oxidative stress within the maternal brain are not associated with impaired neuronal activity or cognitive performance during pregnancy. However, a previous healthy pregnancy history is associated with rodent learning deficits and reduced anxiety-like behavior, highlighting long-term effects of healthy pregnancy on maternal cognitive function. Future studies examining pathophysiological elevations in maternal brain oxidative stress, such as in pregnancy complications with hypoxic insults, may reveal underlying mechanisms contributing to associated adverse neural and behavioral plasticity in the transition to motherhood.

## DATA AVAILABILITY

Data will be made available upon reasonable request.

## SUPPLEMENTAL MATERIALS

10.6084/m9.figshare.25438894 Supplemental Fig. S1: https://doi.org/10.6084/m9.figshare.25438894.

10.6084/m9.figshare.23982444 Supplemental Fig. S2: https://doi.org/10.6084/m9.figshare.23982444.

10.6084/m9.figshare.23982261 Supplemental Fig. S3: https://doi.org/10.6084/m9.figshare.23982261.

10.6084/m9.figshare.23980191 Supplemental Table S1: https://doi.org/10.6084/m9.figshare.23980191.

## GRANTS

This study was supported by National Institutes of Health Grants R01 HL146562-04S1 (to S.G.) and T32 AG020494 (to S.M.) and American Heart Association Grants 22POST-903250 (to J.L.B.), 22PRE-900431 (to J.J.G.), and 23PRE-1012811 (to S.M.T).

## DISCLAIMERS

The content is solely the responsibility of the authors and does not necessarily represent the official views of the National Institutes of Health.

## DISCLOSURES

No conflicts of interest, financial or otherwise, are declared by the authors.

## AUTHOR CONTRIBUTIONS

J.L.B., S.G., and R.L.C. conceived and designed research; J.L.B., E.N.W., J.J.G., S.M., S.M.T., and N.R. performed experiments; J.L.B., E.N.W, and E.V. analyzed data; J.L.B., S.G., and R.L.C. interpreted results of experiments; J.L.B. prepared figures; J.L.B. drafted manuscript; J.L.B., E.N.W., J.J.G., S.M., S.M.T., N.R., E.V., S.G., and R.L.C. edited and revised manuscript; J.L.B., E.N.W., J.J.G., S.M., S.M.T., N.R., E.V., S.G., and R.L.C. approved final version of manuscript.
